# Volumes of cancer surgery for breast, colorectal and ovarian cancer 1992–97: is there evidence of increasing sub-specialization by surgeons?

**DOI:** 10.1054/bjoc.2001.1794

**Published:** 2001-05

**Authors:** K Jolly, J Parry, A Rouse, A Stevens

**Affiliations:** Department of Public Health and Epidemiology, University of Birmingham, Edgbaston, Birmingham, B15 2TT, UK

**Keywords:** breast neoplasms, colorectal neoplasms, ovarian neoplasms, volume of clinical activity

## Abstract

The ‘Calman–Hine Report’ (1995) recommended that cancer surgery should be limited to ‘high-volume’ consultants. Through an analysis of 5 years of Hospital Episode Statistics for the West Midlands region (1992–1997), we have investigated whether there is evidence of increasing numbers of patients with breast, colorectal or ovarian cancer being treated by high throughput, i.e. sub-specialist surgeons, who carry out more than a threshold level of primary cancer resections annually. The proportion of cases treated by the high-volume breast, colorectal and ovarian cancer surgeons increased annually during the 5 years. The absolute number of consultant firms who undertook breast cancer resections reduced during the 5 years; but the number doing colorectal and ovarian surgery increased. Throughout the 5 years, half of the ovarian cancer resections were carried out by consultant firms who did very few procedures – less than 5 of these procedures annually. The relatively high case-load, the elective nature of breast cancer surgery and an early policy change have undoubtedly facilitated the move towards sub-specialization. The weaker trends for colorectal and ovarian cancer surgery suggest continued monitoring is required to ensure that there is a reduction in the proportion of people treated by surgeons who undertake few cancer resections annually. © 2001 Cancer Research Campaign www.bjcancer.com


*British Journal of Cancer* (2001) **84**(10), 1308–1313
© 2001 Cancer Research Campaign
doi: 10.1054/ bjoc.2001.1794, available online at http://www.idealibrary.com on IDEAL®

http://www.bjcancer.com

### Volumes of cancer surgery for breast, colorectal and ovarian cancer 1992–97: is there evidence of increasing sub-specialization by surgeons?

**K Jolly, J Parry, A Rouse and A Stevens**

Department of Public Health and Epidemiology, University of Birmingham, Edgbaston, Birmingham, B15 2TT, UK

**Summary** The ‘Calman–Hine Report’ (1995) recommended that cancer surgery should be limited to ‘high-volume’ consultants. Through an analysis of 5 years of Hospital Episode Statistics for the West Midlands region (1992–1997), we have investigated whether there is evidence of increasing numbers of patients with breast, colorectal or ovarian cancer being treated by high throughput, i.e. sub-specialist surgeons, who carry out more than a threshold level of primary cancer resections annually. The proportion of cases treated by the high-volume breast, colorectal and ovarian cancer surgeons increased annually during the 5 years. The absolute number of consultant firms who undertook breast cancer resections reduced during the 5 years; but the number doing colorectal and ovarian surgery increased. Throughout the 5 years, half of the ovarian cancer resections were carried out by consultant firms who did very few procedures – less than 5 of these procedures annually. The relatively high case-load, the elective nature of breast cancer surgery and an early policy change have undoubtedly facilitated the move towards sub-specialization. The weaker trends for colorectal and ovarian cancer surgery suggest continued monitoring is required to ensure that there is a reduction in the proportion of people treated by surgeons who undertake few cancer resections annually. © 2001 Cancer Research Campaign http://www.bjcancer.com

**Keywords**: breast neoplasms; colorectal neoplasms; ovarian neoplasms; volume of clinical activity

In 1995, the Expert Advisory Group on Cancer to the Chief Medical Officers of England and Wales published recommendations (the ‘Calman–Hine’ Report) on the management of cancer (Expert Advisory Group on Cancer, 1995). The report argued that, at that time, patients were being admitted to units that were not adequately equipped to provide the full range of diagnostic and therapeutic interventions, with the consequence that the quality of care was compromised, and varied substantially across the country. One of the principal problems identified was insufficient concentration of service provision, so limiting the opportunity for clinical staff to sub-specialize.

Central to the conclusions of the Expert Advisory Group’s Report is the recommendation to limit cancer surgery to site-specialized ‘high-volume’ consultants working in multi-disciplinary teams in designated cancer units and centres. Research evidence of improved outcomes in patients treated by specialist or high-volume consultants exists for breast cancer, but is less clear for colorectal and ovarian cancers (Sainsbury et al, 1995; Gillis and Hole, 1996). A number of studies have explored the relationship between surgical volume of work and outcome from colorectal cancer, but although variation in practice and outcome have been observed (Fielding et al, 1980; Phillips et al, 1984; McCardle and Hole, 1991; Mella et al, 1997) a clear and consistent association with throughput is not apparent with some studies suggesting a relationship (Hermanek and Hohenberger, 1996; Porter et al, 1998) and others not (Sagar et al, 1996; Parry et al, 1999). With regard to ovarian cancers, an association with throughput again is not clear, although improved outcomes have been noted among specialist gynaecologists compared to general surgeons (Nguyen et al, 1993; Junor et al, 1994; Kehoe et al, 1994).

Despite the availability of a robust evidence base, the Calman–Hine Report advocated service centralization as a means of improving patient outcome. Thus, if the recommendations have been implemented successfully, we would have expected there would have been more surgeons undertaking a sufficient volume of work to maintain sub-specialization skills in 1997 than in 1992. However, given the phased nature of the Calman–Hine implementation process with breast, colorectal and lung cancers being chosen as the initial sites for service reorganization, followed more recently by recommendations for the management of gynaecological malignancies, it might be anticipated that sub-specialization would have occurred at different speeds for different tumours. The degree to which this has occurred is likely to be of central interest to the new Commission for Health Improvement in their monitoring of progress towards the implementation of the recommendations in the Calman–Hine Report.

We have therefore analysed changing patterns for the surgical management of breast, colorectal and ovarian cancer in the West Midlands region during the period 1992–1997. Specifically the 2 questions addressed by the study are whether between 1992 and 1997 (i) an increasing proportion of cases were treated by ‘high-volume’ consultant firms; and (ii) fewer (and therefore fewer low-volume) consultant firms were undertaking primary resective cancer surgery.

### METHODS

### Study setting and population

We based our study on the West Midlands region of the NHS. The region serves a population of 5.3 million residents which

*Received 23 October 2000*
*Revised 26 January 2001*
*Accepted 20 February 2001*

*Correspondence to:* K Jolly comprises more than 10% of the population of England and Wales. The region's demographic composition and health service provision is typical of England and Wales.

### Data abstraction and case definition

We used the Hospital Episode Statistics (HES) dataset to identify records of patients who (i) were admitted to NHS hospitals between 01/04/92 and 31/03/97, (ii) had an admission diagnostic code for breast, ovarian or colorectal cancer, and (iii) underwent a surgical resection the nature of which was compatible with the primary excision of the tumour. For example, for ovarian cancer this included all excisions of the ovary, adnexae and uterus but excluded non-specific laparotomy or stoma formation without ovarian resection. We were unable to extend the study period beyond 1997 due to changes in the HES dataset which precluded identification of individual consultants. For all 3 sites, patients managed non-surgically by radiotherapy and/or chemotherapy only were excluded from the analysis. Details of the diagnostic and procedural codes we used are shown in Table 1. For each case included in the analysis, information on date of operation and consultant code were abstracted from the HES dataset.

### Case volume definition and selection of thresholds

We defined the surgical volume of each consultant firm as the number of procedures which involved primary resection of the tumour (Table 1) undertaken each year (1 April to 31 March). The term 'procedure' is used throughout the text to refer to primary resections. Throughput was calculated for the consultant firm rather than individual clinician because HES only permits the identification of the lead consultant responsible for care.

For each site a series of throughput groups were created. For breast cancer we defined a high-volume consultant firm after reviewing the evidence on outcomes for surgery and taking into account College and national recommendations on minimum acceptable workloads for surgeons (BASO, 1995; Sainsbury et al, 1995). For colorectal and ovarian cancers, the literature was reviewed for evidence of appropriate thresholds but no robust information was available and thus we selected cut-offs that reflected the incidence of disease. For breast and colorectal cancer these were: less than 10 procedures per year, 10–29, 30–49, and 50 or more. For ovary the groups were: 1, 2–4, 5–9 and 10 or more per year, reflecting the lower incidence of disease. The proportion of cases managed by consultant firms undertaking different volumes of work, and the number of consultant firms in each throughput group were calculated for each year of the study. For breast cancer high volume consultant firms were those undertaking 30 or more procedures per year, for colorectal cancer the threshold was 50 procedures, and for ovarian cancer 10 procedures per year.

### Statistical analysis

We used Excel and SPSS for Windows™ to store and analyse data. A Chi-squared test for trend (ordered) was used to establish whether there was a trend over time in the proportion of cases treated by the high volume consultant firms. All tests of significance were at the 5% two-sided level and were focussed on the threshold for high-volume consultant firms to avoid multiple testing.

### RESULTS

Over the 5-year period, there were 26 351 admissions to hospital with both a diagnostic code for breast cancer and an OPCS-coded procedure in hospital, 14 227 of which had a surgical procedure consistent with primary resection of their disease. The remaining admissions were for radiotherapy, chemotherapy or procedures not related to breast cancer. Of the admissions with a diagnosis of colon or rectal cancer, 12 803 had a colonic or rectal resection. There were 1326 excisions of the ovary on patients with ovarian cancer (Table 1).

### Patient data

Figures 1–3 show the proportion of cases each year who were treated by consultant firms that undertook different volumes of site-specific resective surgery. For breast cancer there is a clear divergent trend between the groups over the 5-year period. The

**Table 1** Diagnostic and procedural codes used to select cases of ovarian, colorectal and breast cancer which were treated by potentially curative surgery, with numbers identified, 1992–97

| | Breast cancer | Colorectal cancer | Ovarian cancer |
|---|---|---|---|
| ICD-9 diagnoses | 174 | 153<br>154 | 183 |
| ICD-10 diagnoses | C50* | C18*<br>C19X<br>C20X<br>C21* | C56X<br>C57 |
| OPCS procedure codes | B27*–B28* | H04*–H17*<br>H33*–H34*<br>H40*–H41* | Q07*–Q08*<br>Q22*–Q24* |
| Number of admissions with diagnosis of breast cancer, and any procedure code | 26 351 | 26 265 | 5763 |
| Number of admissions with diagnosis, and a procedure code for resective breast surgery | 14 227 | 12 803 | 1326 |

*© 2001 Cancer Research Campaign*

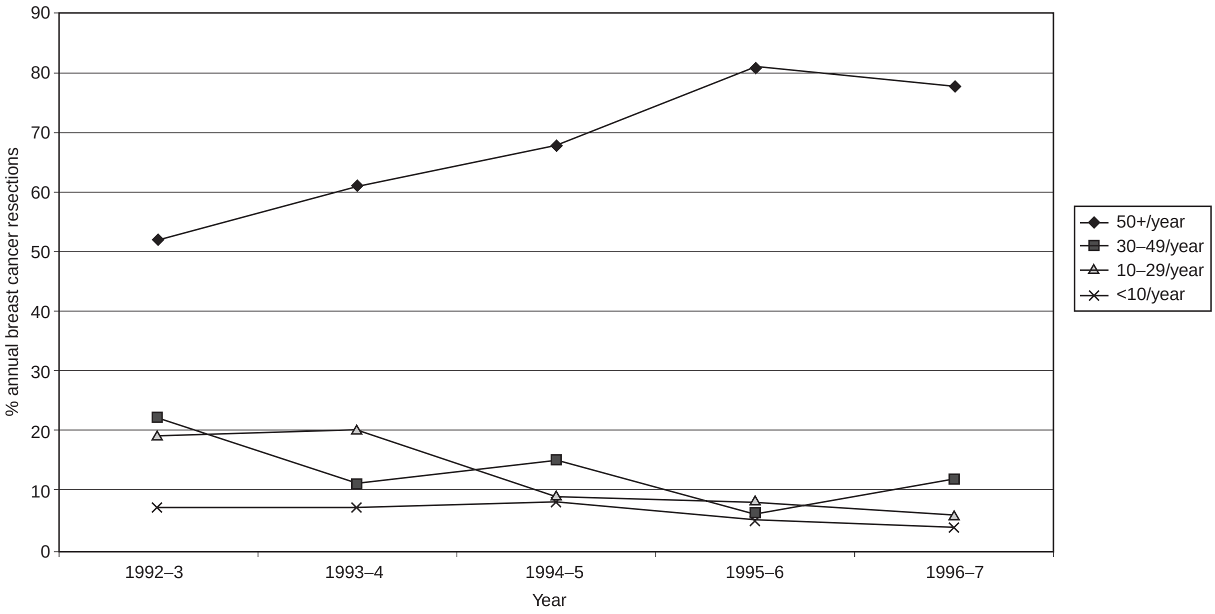

**Figure 1** Proportion of breast cancer operations carried out by consultant firms undertaking different volumes of breast surgery

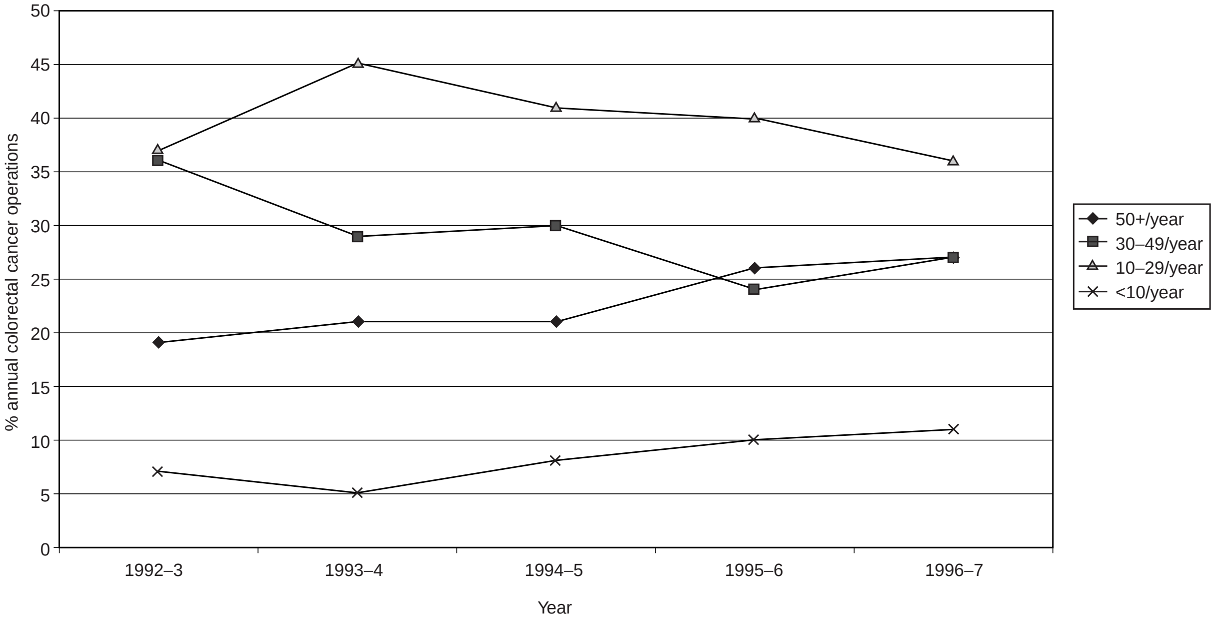

**Figure 2** Proportion of colorectal cancer operations carried out by consultant firms undertaking different volumes of colorectal cancer surgery proportion of cases managed by the highest volume (> 50 cases) consultant firms increased from 52% in 1992/93 to 78% in 1996/97 with a concomitant decrease in the proportion of cases treated by consultant firms operating on < 10, 10–29 and 30–49 cases per year. A significant but much less marked trend was noted for ovarian cancer with a gradual increase, but still a minority, of higher-volume cases. For colorectal cancer however the picture was mixed. There was an increase in the proportion of cases managed by both the highest (50 cases or more) and lowest (< 10) volume consultant firms, with workload shifting from those firms managing 10–49 cases per year.

The overall proportion of cases treated by high-volume consultant firms was substantially greater for breast cancer (90% in 1996/97) than for either colorectal (27%) or ovarian (11%) disease. However, for all 3 sites, the proportion of cases managed by a high-volume consultant firm increased significantly over the study period (breast $\chi^2_{(trend)(1\ df)} = 438$; $P < 0.001$; colorectal $\chi^2_{(trend)(1\ df)} = 58$; $P < 0.001$; and ovary $\chi^2_{(trend)(1\ df)} = 15$; $P < 0.001$).

### Consultant firm data

Table 2 shows details of the number of consultant firms that undertook differing volumes of resective surgery annually. The overall numbers of consultant firms undertaking breast cancer surgery reduced in 1995–96 and then more markedly in 1996–97. In contrast the numbers of consultant firms which undertook surgical

 *© 2001 Cancer Research Campaign*

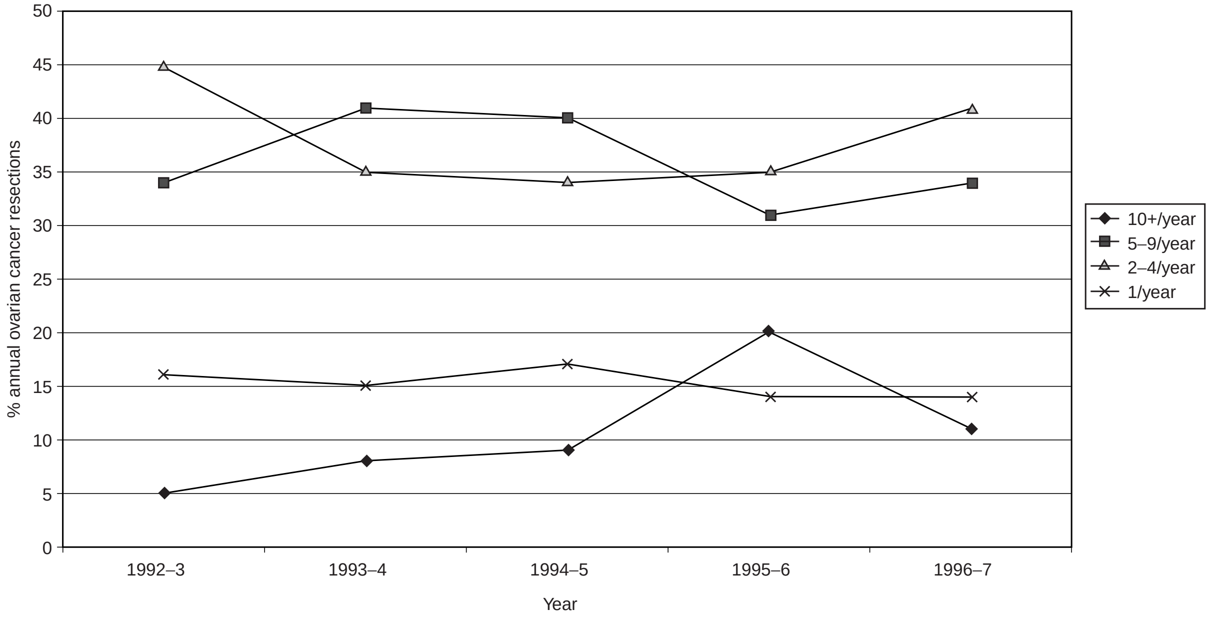

**Figure 3** Proportion of ovarian cancer operations carried out by consultant firms undertaking different volumes of ovarian surgery

**Table 2** Number of consultant firms undertaking differing volumes of breast, colorectal and ovarian cancer resections, 1992–97

| | Number of consultants undertaking different annual volumes of cancer surgery | | | | | | | | | |
|---|---|---|---|---|---|---|---|---|---|---|
| **Annual number of cancer resection** | **1992–3** | **%** | **1993–4** | **%** | **1994–5** | **%** | **1995–6** | **%** | **1996–7** | **%** |
| Breast | | | | | | | | | | |
| <10 | 47 | 42 | 56 | 50 | 70 | 60 | 57 | 54 | 41 | 49 |
| 10–29 | 32 | 29 | 29 | 26 | 16 | 14 | 16 | 15 | 10 | 12 |
| 30–49 | 16 | 14 | 8 | 7 | 10 | 9 | 5 | 5 | 9 | 11 |
| 50+ | 17 | 15 | 20 | 18 | 21 | 18 | 27 | 26 | 24 | 29 |
| total | 112 | 100 | 113 | 100 | 117 | 100 | 105 | 100 | 84 | 100 |
| Colorectal | | | | | | | | | | |
| <10 | 62 | 42 | 56 | 38 | 74 | 47 | 78 | 48 | 101 | 56 |
| 10–29 | 52 | 35 | 64 | 43 | 56 | 35 | 57 | 35 | 52 | 29 |
| 30–49 | 26 | 18 | 20 | 14 | 20 | 13 | 16 | 10 | 17 | 9 |
| 50+ | 7 | 5 | 8 | 5 | 8 | 5 | 10 | 6 | 10 | 6 |
| total | 147 | 100 | 148 | 100 | 158 | 100 | 161 | 100 | 180 | 100 |
| Ovary | | | | | | | | | | |
| 1 | 36 | 40 | 40 | 44 | 38 | 46 | 47 | 44 | 41 | 41 |
| 2–4 | 41 | 46 | 32 | 35 | 27 | 33 | 40 | 37 | 42 | 42 |
| 5–9 | 12 | 13 | 17 | 19 | 15 | 18 | 17 | 16 | 15 | 15 |
| 10+ | 1 | 1 | 2 | 2 | 2 | 2 | 4 | 4 | 2 | 2 |
| total | 90 | 100 | 91 | 100 | 82 | 100 | 108 | 100 | 100 | 100 |

resections for colorectal and ovarian cancer increased over the period. For all 3 cancers however, a large proportion of consultant firms was still undertaking very low numbers of procedures at the end of the 5-year period. In ovarian cancer in particular, in excess of 80% of ovarian cancer consultant firms who undertake ovarian cancer surgery at all, do less than 5 ovarian cancer resections each per year.

### DISCUSSION

The results from this study suggest that between 1992 and 1997, the proportion of cancer cases who were treated by consultant firms undertaking high volumes of work increased significantly. We would expect this to translate into better outcomes for patients. However, the patterns of change were not the same across cancer sites. In breast cancer there was a clear shift in patient workload to the high-volume consultant firms from those undertaking primary resections less frequently. A similar pattern was noted for ovary although it was considerably less marked, and even by the end of the study period the proportion of cases managed by a high-volume consultant firm was only 11%. For colorectal cancer high-volume consultant firms undertook an increasing proportion of caseload. However an increase in the proportion of cases managed by low-volume consultant firms was also noted. The

*© 2001 Cancer Research Campaign*

evidence to support an association between throughput and outcome is stronger for breast cancer (Sainsbury et al, 1995) than for either colorectal or ovarian tumours. There is some suggestion in the literature that any association between consultant throughput and outcome is more marked for rectal than for colon cancer (Hermanek and Hohenberger, 1996; Porter et al, 1998), although a more recent study from the United States has suggested that at a hospital level, increased throughput is also associated with improved outcome from colon cancer (Schrag et al, 2000). Although cognisant of the potential advantages of considering colon and rectal cancers separately, we made no attempt to dissaggregate tumours of the large bowel into ‘colon’ and ‘rectum’ because the Calmin–Hine recommendations treat colorectal cancer as a single disease entity.

This study has described management trends in a population of 5.3 million residents with demographic characteristics and service provision similar to elsewhere in England. However, one factor which might affect the validity of our findings is the completeness and accuracy of the HES data. This has been studied in the past with the most recent figures of 98% completeness for key fields (McKee, 1993). Information on the West Midlands HES reported a 1.4% non-completion rate for the main diagnostic codes in 1994–95 (Wilson, 1997). Approximately half of all the admissions in this series with a diagnosis of breast, colorectal or ovarian cancer had no associated resective procedure. It is probable that these admissions were for non-surgical reasons. However, it is also possible that the absence of a surgical procedure reflects incomplete coding within Trusts. To estimate whether this potential coding deficiency could affect our findings, we compared the number of cases in our hospitalized series with incidence data from the West Midlands Regional Cancer Registry. This data suggests that we were successful in identifying at least 89% of breast, 82% of colorectal and 71% of ovarian cancer resections. Thus we believe that we identified the majority of resective procedures carried out on patients with these cancers. If coding deficiencies have led to an under-ascertainment of cases then we do not believe there is a systematic bias in our results, as the proportion of cancer cases with procedure codes in our dataset does not vary significantly between high- and low-volume hospitals.

In this study we used a tight definition for the surgical procedure, and thus also for the estimation of volume of work. To be included in the analysis cases had to have an OPCS code consistent with the admission diagnosis and with resection of the tumour. In this way we believe that we have only identified the primary surgical procedure. However two misclassification errors are possible. Firstly, it is inevitable that we will have included some procedures for tumour recurrence and not primary resection. If resection of recurrent disease is more likely to be undertaken by a specialist consultant firm, then the inclusion of some of these cases in our series will have overestimated the throughput of high-volume relative to lower-volume consultant firms, i.e. the increase in proportion of cases managed by high-volume consultant firms could be due to change in referral pattern for recurrent rather than primary disease. Secondly, we will have underestimated the total amount of cancer surgery done by consultant firms, as a proportion of cases will present at an advanced stage and be unsuitable for resective surgery. If the proportion of advanced, non-resectable cases is greater among the workload of low-volume surgeons then the overall shift from low to high groups reported here will, in reality, be less marked and the proportion of cases seen by high-volume consultant firms over-estimated, i.e. we have over-estimated the effectiveness of the implementation process.

The data used in this study were derived from the HES dataset. We were unable to extend the study period beyond 1997 due to changes in the HES dataset which precluded identification of individual consultants. An option to circumvent this difficulty would be to use probablistic linkage techniques to link HES data with that of the regional cancer registry to ascertain the identity of the consultant responsible for care (Pollock and Vickers, 1998). However such linkage requires information of explicit patient identifiers (date of birth, sex and postcode address). During the period when this study was being undertaken, there were substantial concerns with regard to the use of cancer registry data and patient consent – concerns that have only been put on hold at present due to an interim (and temporary) statement from the General Medical Council. In view of this, linkage between the HES dataset and information held by the regional cancer registry was not feasible.

The Calman–Hine report was published in 1995, with national guidance on the management of breast and colorectal cancers produced in 1995, 1996 and 1997 (BASO, 1995; Royal College of Surgeons, 1996; NHS Executive, 1997). High-volume consultant firms now manage over 90% of breast cancer cases, and there are clear and consistent shifts in workload from low- to high-volume consultant firms. This, we believe is due in part to the appointment of specialist breast surgeons in the early 1990s even before the implementation of Calman–Hine, and also to the elective nature of breast cancer surgery. In contrast, about one-third of patients with colorectal cancer present non-electively, often with bowel obstruction that requires immediate surgery by the surgical team on-call. Thus although there is evidence of a shift in workload from medium- to high-volume consultant firms (Figure 2) the proportion of cases treated by very low-volume consultant firms has also increased. The increase in the numbers of consultant firms doing very low annual volumes of resective surgery noted in this study may therefore have arisen as a consequence of the expansion of consultant numbers. If this is the case, and the cases managed by very low-volume consultant firms are emergencies, then to achieve a reduction in the proportion of colorectal cancer cases managed by very low-volume consultant firms would necessitate a reorganization of on-call arrangements or the time of surgery delayed until the specialist colorectal cancer surgeon is available. This is already being practiced in some hospitals nationally. Further work to ascertain whether the low-volume surgery is made up of emergency cases would be informative.

The comparative rareness of ovarian cancer necessitates referral of all cases to a limited number of sub-regional specialists working in cancer centres, a process advocated by the recently published guidelines on the management of gynaecological cancers (NHS Executive, 1999). This is the first cancer site which has required a solution beyond the reorganization of referral patterns within an individual hospital. It is not surprising therefore that up until the present, although some shifts in workload patterns have been noted, only a minority of cases have been treated by high-volume consultant firms. Furthermore gynaecological cancers were not among the initial tranche of cancers targeted by the Calman–Hine reorganization process and guidance for the management of gynaecological cancers has only been produced within the last 12 months. It is unsurprising therefore that only limited progress has been made. However, as with other cancer sites, for example head and neck and upper gastrointestinal disease, the implementation of the Calman–Hine recommendation for gynaecological cancers

© 2001 Cancer Research Campaign requires reorganization and changes in referral patterns not only within but also between Trusts. This process has already begun in the West Midlands region with the establishment of cancer networks. Given the need to monitor progress towards these service delivery goals, it is unfortunate that since 1997 information on consultant identity is no longer routinely available with the HES activity data in the West Midlands. The methods used in this study therefore cannot be replicated in future to explore subsequent trends in subspecialization. The importance of robust systems for the monitoring of cancer care need to be addressed by the Centre for Health Improvement, and other methods including the use of cancer registry data, explored. This later approach would have the advantage of being based on patients, rather than activity data and contains information on outcome, which could make an important contribution to monitoring cancer services.

### ACKNOWLEDGEMENTS

We acknowledge the contribution of Richard Wilson in providing us with data from the HES database.

### REFERENCES

British Association of Surgical Oncologists (BASO) (1995) Guidelines for the mangement of symptomatic breast disease in the United Kingdom. *Eur J Surg Oncol* **21**(A)

Expert Advisory group on Cancer to the Chief Medical Officiers of England and Wales (1995) *A policy framework for commissioning cancer services*. Department of Health

Fielding LP, Stewart-Brown S, Blesovsky L and Kearney G (1980) Anastomotic integrity after operations for large-bowel cancer: a multicentre study. *BMJ* **281**: 411–414

Gillis CR and Hole DJ (1996) Survival outcome of care by specialist surgeons in breast cancer: a study of 3786 patients in the West of Scotland. *BMJ* **312**: 145–148

Hermenek P, Hohenberger W (1996) The importance of volume in colorectal cancer surgery. *Eur J Surg Oncol* **22**: 213–215

Junor EJ, Hole DJ and Gillis CR (1994) Management of ovarian cancer: referral to a multidisciplinary team matters. *Br J Cancer* **70**: 363-370

Kehoe S, Powell J, Wilson S and Woodman C (1994) The influence of operating surgeon's specialisation on patient survival in ovarian cancer. *Br J Cancer* **70**: 1014–1017

McCardle CS and Hole D (1991) Impact of variability among surgeons on postoperative morbidity and ultimate survival. *BMJ* **302**: 1501–1505

Mella J, Biffin A, Radcliffe AG, Stamatakis JD and Steele RJC (1997) Population-based audit of colorectal cancer management in two UK health regions. *Br J Surg* **84**: 1731–1736

NHS Executive (1997) Guidance for purchasers. Improving outcomes in breat cancer. The manual. Department of Health

NHS Executive (1999) Improving outcomes in gynaecological cancers. The Manual. Department of Health

Nguyen HN, Averette HE and Hoskins W, Penalver M, Sevin B and Steren A (1993) National survey of ovarian carcinoma, Part V. *Cancer* **72**: 3663–3670

Parry JM, Collins S, Mathers J, Scott NA and Woodman CBJ (1999) The influence of work volumes on outcome of patients with colorectal carcinoma'. *British Journal of Surgery* **86**: 475–481

Phillips RKS, Hittinger R, Blesovsky L, Fry JS and Fielding LP (1984) Local recurrence following 'curative' surgery for large bowel cancer: I. The overall picture. *Br J Surg* **71**: 12–16

Pollock AM and Vickers N (1998) Trends in colorectal cancer care in Southern England, 1989–1993: using HES data to inform Cancer Services reviews. *J Epidemiol Community Health* **52**: 433–438

Porter GA, Saskolne CL, Yakimets WW and Newman SC (1998) Surgeon-related factors and outcome in rectal cancer. *Ann Surg* **227**: 157–167

Sagar PM, Hartley MN, MacFie J, Taylor BA and Copeland GP (1996) Comparison of individual surgeon's performance: risk adjusted analysis with POSSUM scoring system. *Dis Colon Rectum* 654–658

Sainsbury R, Haward B, Rider L, Johnston C and Round C (1995) Influence of clinician workload and patterns of treatment on survival from breast cancer. *Lancet* **345**: 1265–1270

Schrag D, Cramer LD, Bach PB, Cohen AM, Warren JL and Begg CB (2000) Influence of hospital procedure volume on outcomes following surgery for colon cancer. *JAMA* **284**: 3028–3035

The Royal College of Surgeons of England and the Association of Coloproctology of Great Britain and Ireland (1996) Guidelines for the management of Colorectal cancer

Wilson R (1997) Utilisation of routinely collected data in health services research: a study of their application across the West Midlands Region of the National Health Service. University of Birmingham

*© 2001 Cancer Research Campaign*
